# Examining Associations of Environmental Characteristics with Recreational Cycling Behaviour by Street-Level Strava Data

**DOI:** 10.3390/ijerph14060644

**Published:** 2017-06-15

**Authors:** Yeran Sun, Yunyan Du, Yu Wang, Liyuan Zhuang

**Affiliations:** 1 Urban Big Data Centre, School of Social and Political Sciences, University of Glasgow, Glasgow G12 8RZ, UK; 2 State Key Laboratory of Resources and Environmental Information System, Institute of Geographic Sciences & Natural Resources Research, Chinese Academy of Sciences, Beijing 100101, China; duyy@lreis.ac.cn; 3 Urban Studies, School of Social and Political Sciences, University of Glasgow, Glasgow G12 8RS, UK; Yu.Wang@glasgow.ac.uk (Y.W.); l.zhuang.1@research.gla.ac.uk (L.Z.)

**Keywords:** cycling, crowdsourced geographic information, Strava, street level, big data

## Abstract

Policymakers pay much attention to effectively increasing frequency of people’s cycling in the context of developing sustainable and green cities. Investigating associations of environmental characteristics and cycling behaviour could offer implications for changing urban infrastructure aiming at encouraging active travel. However, earlier examinations of associations between environmental characteristics and active travel behaviour are limited by low spatial granularity and coverage of traditional data. Crowdsourced geographic information offers an opportunity to determine the fine-grained travel patterns of people. Particularly, Strava Metro data offer a good opportunity for studies of recreational cycling behaviour as they can offer hourly, daily or annual cycling volumes with different purposes (commuting or recreational) in each street across a city. Therefore, in this study, we utilised Strava Metro data for investigating associations between environmental characteristics and recreational cycling behaviour at a large spatial scale (street level). In this study, we took account of population density, employment density, road length, road connectivity, proximity to public transit services, land use mix, proximity to green space, volume of motor vehicles and traffic accidents in an empirical investigation over Glasgow. Empirical results reveal that Strava cyclists are more likely to cycle for recreation on streets with short length, large connectivity or low volume of motor vehicles or on streets surrounded by residential land.

## 1. Introduction

In the context of developing sustainable and green cities, selecting active travel (cycling and walking) instead of inactive travel (travel by motorized vehicles) to work is encouraged. This produces health benefits by means of enhancing physical activity, improves traffic efficiency and reduces air pollution [1,2,3,4,5,6,7,8,9,10,11,12]. Effectively increasing frequency of people’s cycling by changing urban infrastructure interests policymakers. Earlier studies revealed that cycling behaviour tends to be affected by environmental characteristics, including population density, land use mix and cycling facilities [13,14,15,16,17,18,19,20,21,22,23,24,25,26,27,28,29,30,31,32,33,34,35,36,37]. For instance, some studies uncovered that usage of bicycles is positively associated with high population density [36,37] and high levels of public transit accessibility [23,26,28], high street density and connectivity [34,35], and a high land use mix [28,35]. At the same time, usage of bicycles is negatively affected by high motorized traffic volume [23,25], low cycling safety [20,27,33], bad weather [20,27] and long commute distance or time [20,31]. Moreover, improving cycling infrastructure (e.g., bicycle lanes) is likely to increase ridership [13,18,19,21,23,24,29,30,32,35].

However, examining the impact of environmental characteristics on cycling behaviour is limited by low spatial granularity and coverage of traditional data. Travel survey data tend to have a low spatial granularity as the geographic level of travel survey data is usually census tract, whilst traffic count data have a high spatial granularity but a low spatial coverage as traffic counts points are usually located on major roads rather than minor roads. Volunteer geographic information (VGI) and crowdsourced geographic information (CGI) offer an opportunity to determine fine-grained mobility and travel patterns of people [38,39,40,41]. VGI is geographic information (GI) voluntarily contributed by a crowd (mainly internet users), whilst CGI is GI actively and passively contributed by a crowd [42]. In very recent years, Strava Metro data have become a new data source for cycling studies [40,41,43,44]. Strava Metro data are produced from users’ GPS traces that are uploaded to Strava. To protect users’ privacy, users’ GPS traces are anonymized and aggregated to streets. As a consequence, hourly, daily or annual cycling volumes in each street are available in Strava Metro data. Some studies suggest that crowdsourced data might be a good proxy for estimating daily cycling volumes by comparing cyclist counts from Strava data and traffic count data [44,45]. This provides further support that Strava Metro data enable better examination of associations of environmental characteristics and cycling behaviour due to a large spatial scale (i.e., street level), a large temporal scale (i.e., secondary level) and a potential proxy for real cycling volume.

On the other hand, Strava Metro data also indicate the purpose (commuting or recreational) of cycling activities [40]. Compared to regular cyclists, Strava cyclists are more likely to cycle for recreation [46]. As travel survey data always focus on commute cycles, Strava Metro data offer a good opportunity for studies of recreational cycling behaviour [40]. Moreover, to the best of our knowledge, street-level recreational cycling behaviour is not well discussed due to a lack of street-level cycling data associated with cycling purpose in the past. Recent studies explored the effects of environmental characteristics on recreational cycling behavior; however, some important environmental characteristics, e.g., traffic volume and traffic accidents, have not been considered [40,41]. Therefore, in this study we made use of Strava Metro data to examine associations of environmental characteristics (i.e., population density, employment density, road length, road connectivity, proximity to public transit services, land use mix, proximity to green space, volume of motor vehicles and traffic accidents) and recreational cycling behaviour. Based on an empirical investigation in Glasgow, we discussed the effects of environmental characteristics on recreational cycling behaviour.

The remainder of this paper is organised as follows. Section 2 introduces the research data including Strava Metro data and environmental characteristics data, as well as measures of environmental characteristics. Section 3 describes the empirical results, and finally Section 4 presents the conclusion and provides recommendations for future work.

## 2. Materials and Methods

Section 2.1 introduces the data and study area, and Section 2.2 and Section 2.3 present how we measure environmental characteristics and recreational cycling behaviour at the street level.

### 2.1. Data and Study Area

#### 2.1.1. Strava Metro Data

Strava (San Francisco, CA, USA) consists of a mobile app and a website, allowing users to track their rides, runs, walks and hikes on a smartphone or another GPS device. The Strava app records GPS traces for each ride, run, walk or hike. Those GPS-tracked activities recorded by the Strava app can be uploaded by users to Strava’s website. Users are able to add titles and tags to describe their trips and also use a “commute” flag to indicate riding journeys to or from work. Strava data can be considered as “big data” since (1) Strava’s database comprises nearly a trillion GPS points globally and is growing by over 8 million activities every week [47], and (2) Strava’s database is not structured as a common dataset. To build a user friendly data format and protect user privacy, Strava Metro anonymizes and aggregates activity data from Strava’s millions of users [48]. Strava Metro is a suite of data services that aims to produce state-of-the-art spatial data products and services to make cycling, running, and walking in cities better [48]. Apart from the “commute” flag, textual information containing keywords such as “To Work” or “Commute To” and distance or time of the GPS trace is used to distinguish commute activities [47].

**Figure 1:**
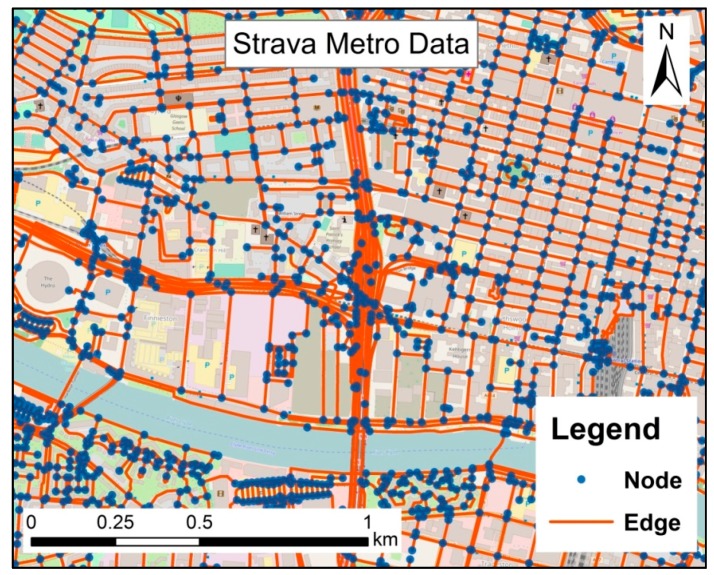
Edges and nodes of Strava Metro data (Basemap: OpenStreetMap, licensed under the Open Database License).

The Urban Big Data Centre, UK, publicly provides a Strava Metro dataset to researchers [49]. This dataset contains cycling activities contributed by Strava users within the Glasgow Clyde Valley Planning area (including Glasgow City and seven council areas) in 2015. This dataset contains three sub sets in three formats: Streets (Edges), Nodes and Origin-Destination (see [48]). Both the Streets and Nodes sets are created based on a road network which is extracted from OpenStreetMap. The Streets set contains all edges of the street network, while the Nodes set contains all nodes of the street network. Each edge represents a street and each node represents an intersection of streets in the road network (see Figure 1). Table 1 lists the attributes of edges, including cycling volume in each edge (street) at a specific time. Note that the time granularity is at the minute level.

Additionally, the dataset contains a file that offers demographics of the cycling activities and the contributors (see Table 2), including average distance of trip, average time of trip, and user base structure by sex and age. A total of 40% of trips are recreational trips, indicating Strava cyclists are more likely to cycle for recreation than regular cyclists. There are more than 10,000 cyclists, and the vast majority of Strava cyclists are male. Interestingly, the largest group of male cyclists is aged 35–44 whilst the largest group of female cyclists is aged 25–34.

In this study, we focus on cycling trips within Glasgow in 2015. There are 78,714 streets in the Strava Metro road network. Here, we briefly introduce the steps of data preprocessing:

1. We removed records on weekend days (Saturday and Sunday);
2. We counted cycling trips by street and time slot (1-h long, e.g., 7:00–7:59 a.m.);
3. We removed streets with abnormal travel volumes. These streets have a smaller number of all-purpose trips than commuting trips.

#### 2.1.2. Environmental Characteristics Data

To measure environmental characteristics, we employed road network data, population, employment and bus stop data, land use data and green space data, traffic flows and accident data. They are introduced as follows:

Road network data: Being extracted from OpenStreetMap (OSM), the road network offered by the Strava Metro dataset has a better spatial coverage than the road network offered by the Glasgow City Council [50]. Therefore, we use the road network offered by the Strava Metro dataset for characterizing the streets in this study. Basic road types of OSM are motorway, trunk, primary, secondary, tertiary, residential, livingstreet, pedestrian, path, bridleway, cycleway, footway, etc. [51]. For simplicity, we grouped road types into two basic classes: *major* and *minor*. We reclassified road class based on road type and allowed transportation type (motor vehicles, non-motor vehicles or mixed). Accordingly, motorway, trunk, primary, secondary were grouped into *major*, whilst the other types were grouped into *minor*.

Population, employment and bus stop data: Scotland’s 2011 census data were used to offer population and employment data as they provide the most updated employment data at a large scale [52]. The geographic level of the population and employment data is the Output Area [53]. DATA.GOV.UK also offers geo-referenced bus stops across the UK [54].

Land use data and green space data: The land use data employed were provided by the European Environment Agency [55]. We used the most updated land use data that were generated based on aerial images taken in 2009. Land use types contain *Residential*, *Industrial_Commercial_Public*, *Other built-up*, *Leisure facilities*, *Agricultural*, *Forests*, *Water bodies*. Green space data were downloaded from Greenspace Scotland [56]. The green space data contains several types: public park and garden, private gardens, woodland and so forth.

Traffic flows and accident data: The UK Department for Transport offers annual average daily flow (AADF) data covering some major roads in Glasgow [57]. An AADF is the average over a full year of the number of vehicles passing a point in the road network each day. The data provide the number of motor vehicles and the number of cycles. DATA.GOV.UK also offers geo-referenced road accidents in the UK [58]. We used road accidents in 2015.

#### 2.1.3. Comparison of Strava Cycling Volumes and Regular Cycling Volumes

To validate whether Strava metro data could be a good proxy for estimating real cycling volumes, we compared Strava cycling volume data and regular cycling volumes at the street-level. Unlike Strava Metro data which have a large spatial coverage, AADF data cover only 119 links (streets) across Glasgow. Firstly, we matched the links of AADF data with streets from the Strava Metro data based on spatial proximity (a 5-m threshold), road name, start junction name and end junction name. Secondly, we measured the correlation between cycling volume from AADF data and Strava’s annual cycling volume by calculating Pearson’s *R* coefficient. Accordingly, the correlation between real annual daily cycling volume and Strava’s annual cycling volume was 0.83, indicating spatial distribution of Strava cycling volume is fairly proportional to that of real cycling volume on major roads across Glasgow.

### 2.2. Recreational Cycling Behaviour

To characterize recreational cycling behaviour, we measured the dominance of recreational trips on a street by an index: *recreational cycling rate* (RCR), representing the rate of recreational trips during a one-hour time slot (e.g., 7:00–7:59 a.m.) on all workdays in 2015. Suppose *s* is a street, RCR of *s* during the time slot *t* is computed as:

(1) $$RCR\left(s,t\right)\;=\frac{{\mathrm{trip}\_\mathrm{cnt}}^{R}\left(s,t\right)}{{\mathrm{trip}\_\mathrm{cnt}}^{R}\left(s,t\right){\mathrm{trip}\_\mathrm{cnt}}^{C}\left(s,t\right)}$$

where $tri{p_{\mathrm{cnt}}}^{R}\left(s,t\right)$ and $tri{p_{\mathrm{cnt}}}^{C}\left(s,t\right)$ represent the respective number of recreational and commuting trips on street *s* during the time slot *t* on all workdays in 2015.

### 2.3. Environmental Characteristics

Earlier studies investigated associations of environmental characteristics, including population density, land use mix, steep inclines, cycling facilities, volume or mix of motor vehicles, and green space proximity, with cycling behaviour [13,14,15,16,17,18,19,20,21,22,23,24,25,26,27,28,29,30,31,32,33,34,35,36,37]. In this study, we selected environmental characteristics according to the environmental characteristics frequently discussed in earlier studies and present data availability. Specifically, we took account of population density, employment density, land use mix, green space proximity, road length, road connectivity, volume of motor vehicles and traffic accidents. Table 3 lists independent variables, including time of day and environmental variables, considered in this study. *Time of the day* (TOTD) was classified into six categories: Very Early AM Hours (12:00 p.m.–3:59 a.m.), Early AM Hours (4:00 a.m.–5:59 a.m.), AM Peak Hours (6:00 a.m.–8:59 a.m.), Mid-Day Hours (9:00 a.m.–2:59 p.m.), PM Peak Hours (3:00 p.m.–5:59 p.m.), Early Evening Hours (6:00 p.m.–7:59 p.m.), and Late Evening Hours (8:00 p.m.–11:59 p.m.).

#### 2.3.1. Socio-Economic Factors

**Figure 2:**
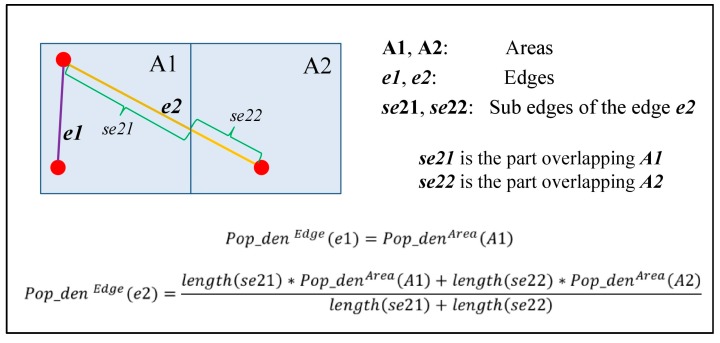
A simple example for calculating the population density of a street.

*Population density* and *employment density* equal the population density and employment density of the Output Area (OA) where the street is located if the street is located completely in this OA; otherwise, *population density* and *employment density* are a weighted mean of population density and employment density of the OAs that overlap the street. Figure 2 shows a simple instance where a street overlaps two different areas (OAs). Suppose *e* is a street and $se_{i}$ (*i* = 1, …, *k*) represents the overlapping part of *e* and a OA. *k* also represents the number of OAs overlapping *e*. The weighted population density and employment density for *e* are calculated as:

(2) $$Po{p_{\mathrm{den}}}^{\mathrm{Edge}}\left(e\right)\;=\frac{\sum_{i=1}^{k}length\left(se_{i}\right)\times Po{p_{\mathrm{den}}}^{\mathrm{OA}}\left(se_{i}\right)}{\sum_{i=1}^{k}length\left(se_{i}\right)}\;Em{p_{\mathrm{den}}}^{\mathrm{Edge}}\left(e\right)\;=\frac{\sum_{i=1}^{k}length\left(se_{i}\right)\times Em{p_{\mathrm{den}}}^{\mathrm{OA}}\left(se_{i}\right)}{\sum_{i=1}^{k}length\left(se_{i}\right)}$$

where $length\left(se_{i}\right)$ represents the length of $se_{i}$, and $Po{p_{\mathrm{den}}}^{\mathrm{OA}}\left(se_{i}\right)$ and $Em{p_{\mathrm{den}}}^{\mathrm{OA}}\left(se_{i}\right)$ represent the respective population density and employment density of the OA where $se_{i}$ is located.

#### 2.3.2. Urban Form Factors

*Distance to city centre* is the distance from the street to the city centre. The centroid of George Square was used to represent the location of the city centre. As a result, *distance to city centre* equals the distance from the street to the centroid of George Square. Technically, imagine a street is a line containing two end vertices and the centroid of George Square is a point. The shortest distance from a point to a line is used to represent the distance from the point to the line. Specifically, the shortest distance from a point to a line is the perpendicular to the line. If a perpendicular cannot be drawn within the two end vertices of the line, then the distance to the closest end vertex is the shortest distance.

*Distance to the nearest bus stop* is the distance from the street to its nearest bus stop. It is used to measure proximity to public transit services.

#### 2.3.3. Road factors

*Road class* is the class of street: *Major* and *Minor*.

*Road length* equals the length of street.

*Connectivity of major road* equals number of major streets (edges) other than the edge itself that is connected to the street (edge).

*Connectivity of minor road* equals number of minor streets (edges) other than the edge itself that is connected to the street (edge).

#### 2.3.4. Land Use and Green Space and Factors

*Land use mix* is mix level of land use in the “local area” of a street. Here, the “local area” of a street is a 10-m square buffer surrounding the street. Here, the buffer size is set to 10 m because (1) the vast majority of parallel roads’ 10-m buffers do not overlap each other; (2) the vast majority of traffic accidents are spatially covered by 10-m buffers of the roads; and (3) the majority of parallel roads’ 10-m buffers overlap more than one land use parcel (polygons) as land use data producer European Environment Agency uses 10 m as the minimum width for linear areas [55]. The area of the “local area” equals street length (m) × 20 m. We used an entropy index to describe the level of land use mix [16,59]. The higher the entropy index, the higher the level of land use mix. Suppose there are *N* land use types, the entropy-based land use mix is represented as:

(3) $$Land\;use\;mix=\;-\overset{N}{\underset{t=1}{\sum }}\frac{LUA\left(t\right)}{LUA\;}\;log_{2}\frac{LUA\left(t\right)}{LUA}$$

where *LUA* (*t*) represents the area of land use type *t* in the 20-m buffer; *LUA* represents the total area of all land use types. In this study, *N* equals 7. The seven land use types are: *Residential*, *Industrial_Commercial_Public*, *Other built-up*, *Leisure facilities*, *Agricultural*, *Forests* and *Water bodies*. The entropy-based land use mix is within the range of 0 to 1, with 0 meaning a single land use type (e.g., all residential) and 1 meaning even distribution of all seven land use types in the 20-m buffer.

*Dominant land use type* (*DLUT*) is the most dominant land use type in the 20-m buffer. For simplicity, we reclassified land use types into four basic classes: *Residential*, *Industrial_Commercial_Public*, *Other built-up* and *Natural*.

*Contiguity to green space* (*CTGS*) is used to indicate if a street is contiguous with any green space. ‘*Yes*’ means that the street is contiguous with a green space, whilst ‘*No*’ means not contiguous. As there are large portions of streets that are contiguous with green spaces, we use *CTGS* instead of distance from the street to its nearest green space.

#### 2.3.5. Traffic-Related Factors

*Volume of motor vehicles* represents the annual average daily volume of motor vehicles on the street.

*Traffic accident density* represents density of traffic accidents within the “local area” of the street. It is used to reflect road cycling safety here. Suppose *e* is a street and *LA*(*e*) is its “local area”, we can compute *traffic accident density* for *e* as

(4) $$Acci\_den\left(e\right)=\frac{Acci\_num\left(LA\left(e\right)\right)}{Area\left(LA\left(e\right)\right)}$$

where *Acci_num*(*LA*(*e*)) represents the number of traffic accidents within the “local area”, and *Area*(*LA*(*e*)) represents the area of the “local area”.

## 3. Results and Discussion

To examine associations of environmental characteristics with RCR, a linear mixed-effects model (also called a linear mixed model) taking account of both fixed effects and random effects was used in this study. For the sake of simplicity, we take account of one random effect, i.e., the intercept, and use each street to represent a group. We assume that different streets might influence the behaviour of cyclists by means of other invisible characteristics (e.g., routes with traffic calming) apart from road characteristics that can be measured by the data available. In this study, 2856 records (24 h × 119 streets) of independent variables (see Table 3) were input into a linear mixed model for RCR. Only 119 streets were considered because those streets were successfully matched with links of AADF data that offer volume of motor vehicles while the other streets were not. Table 4 lists the estimation results for the linear mixed-effects model of RCR. The number of observations is 2856 equal to the number of records, and the number of groups is 119 equal to the number of streets. In Table 4, the coefficient is the coefficient estimated for each independent variable in the fixed effects, and the SE is the standard error for each independent variable; the *p*-value indicates the statistical significance of each independent variable. In this study, a *p*-value below 0.05 means the corresponding independent variable has a statistically significant association with the dependent variable at a 0.05 level. Moreover, a positive coefficient means the corresponding independent variable has a positive association with RCR, while a negative coefficient means the corresponding independent variable has a negative association with RCR.

In regard to the *time of the day*, Strava cyclists are more likely to cycle for recreation during *Very Early AM Hours* (12:00 p.m.–3:59 a.m.), *PM Peak Hours* (3:00 p.m.–5:59 p.m.), *Early Evening Hours* (6:00 p.m.–7:59 p.m.) and *Late Evening Hours* (8:00 p.m.–11:59 p.m.) than they are during the other times of the day. In other words, Strava cyclists are more likely to cycle for recreation from 3 p.m. to late evening.

We examined the impacts of environmental factors on RCR as follows:

First, of the socio-economic factors, neither *residential density* nor *employment density* is significantly associated with RCR. Second, of the urban form factors, neither *distance to city centre* nor *distance to the nearest bus stop* is significantly associated with RCR. Third, of the road factors, *road class* does not have a significant association with RCR, whilst *road length* has a significant and negative association with RCR. This indicates that Strava cyclists are more likely to cycle for recreation on short streets. Both *connectivity of major road* and *connectivity of minor road* are positively and significantly associated with RCR. This indicates that Strava cyclists are more likely to cycle for recreation on streets with large road connectivity. Fourth, of the land use and green space factors, *land use mix* does not have a significant association with RCR, whilst regarding the dominant land use type, only “*Residential*” has a significant and positive association with RCR. This indicates that Strava cyclists are more likely to cycle for recreation on the streets surrounded by residential land than ride on streets surrounded by commercial or industrial land. Surprisingly, *CTGS* “*Yes*” does not have a significant association with RCR. This contradicts our expectation. We expected that *CTGS* “*Yes*” would have a significant and positive association with RCR. The reason might be that not only recreational cyclists but also commuting cyclists like to pass streets close to green space. Fifth, of the traffic-related factors, *volume of motor vehicles* has a significant and negative association with RCR, whilst *traffic accident density* does not have a significant association with RCR.

Above all, Strava cyclists are more likely to cycle for recreation in the afternoon and evening. Of the environmental factors, *road length*, road connectivity (*connectivity of major road* and *connectivity of minor road*), *DLUT* “*Residential*” and *volume of motor vehicles* are significantly associated with RCR, whilst the other factors are not. This indicates that Strava cyclists are more likely to cycle for recreation on streets with short length, large connectivity or low volume of motor vehicles or on streets surrounded by residential land.

Considering that the population structure (gender, age and other socio-economically personal characteristics) between Strava cyclists and regular cyclists is likely to be different, environmental effects on cycling behaviour of Strava cyclists might be different from environmental effects on the cycling behaviour of regular cyclists. More studies need to be done to further explore to what extent Strava cycling data can represent real cycling data spatially and temporally. More importantly, estimating real cycling volume based on Strava cycling volume would increase the potential of Strava Metro data in studies of cycling and health [40].

## 4. Conclusions

This study has examined associations of environmental characteristics with recreational cycling behaviour in Glasgow. Empirical results uncover that Strava cyclists are more likely to cycle for recreation on streets with short length, large connectivity or low volume of motor vehicles or on streets surrounded by residential land.

### 4.1. Limitations

There are some limitations in this study that need to be presented. First, although we empirically proved that Strava cyclists tend to be spatially proportional to real cyclists in Glasgow, real cyclist are only counted on major roads rather than minor roads. Second, to what extent Strava cycling volume is temporally proportional to regular cycling volume in Glasgow is unknown as the AADF data employed do not offer hourly volume of cycles. Third, because the AADF data have a small spatial coverage, only a very small portion of streets over the city were sampled, whilst the others were not due to a lack of volume of motor vehicles. Ideally, a sample consisting of a larger portion of streets over the city would enable a better investigation of environmental effects on cycling behaviour.

### 4.2. Future Works

In future research, some aspects should be considered for further study. First, other potential environmental characteristics such as traffic calming should be considered. Second, we will undertake similar investigations over other cities. We may compare environmental effects in different cities. Third, it is interesting to examine the impact of weather on recreational cycling behaviour, as the temporal granularity of Strava Metro data is high.

## Figures and Tables

**Table 1:** Fields in the Streets file (source: [48]).

Field	Description
Edge_id	Unique and permanent Street ID number for delivery.
Year	Numerical year format (yyyy).
Day	Numerical day format (1–365).
Hour	Numerical hour format (0–24).
Minute	Numerical minute format (0–59).
Count_Ride	Count of all-purpose cycling trips (regardless of unique cyclists) on the section of street for the day, hour and minute.
Commute_Count_Ride	Count of commuting cycling trips (regardless of unique cyclists) on the section of street for the day, hour and minute.
Recreation_Count _Ride	Count of recreational cycling trips (regardless of unique cyclists) on the section of street for the day, hour and minute.

**Table 2:** Demographics of Strava cyclists in 2015.

Statistics								
Athlete ID count (User count)						13,684		
Activity count (Trip count)						287,833		
Commute count (Commute trip count)						174,758		
Recreational count (Recreational trip count)						113,075		
Average distance of trips						24 km		
Average time of trips						81 min		
**Gender**	**Under 25**	**25–34**	**35–44**	**45–54**	**55–64**	**Over 64**	**No Birth Date**	**Total**
**Male**	718	2176	2957	2028	448	73	2812	11,212
**Female**	141	417	346	217	44	2	531	1698

**Table 3:** Independent variables considered in this study.

Variable Type	Indepedent Variables	Type
Temporal factor	Time of the day	categorical
Socio-economic factors	Population density (/ha)	numeric
	Employment density (/ha)	numeric
Urban form factors	Distance to city centre (km)	numeric
	Distance to the nearest bus stop (km)	numeric
Road factors	Road class	categorical
	Road length (km)	numeric
	Connectivity of major road	numeric
	Connectivity of minor road	numeric
Land use and green space factors	Land use mix	numeric
	Dominant land use type	categorical
	Contiguity to green space	categorical
Traffic-related factors	Volume of motor vehicles (k)	numeric
	Traffic accident density (/m square)	numeric

**Table 4:** The estimated linear mixed-effects model for RCR.

	Coefficient	SE	*p*-Value
Intercept	–0.085204	0.04942	0.0848
TOTD “*Very Early AM Hours*”	0.143248	0.01274	<0.0001
TOTD “*Early AM Hours*”	0.015746	0.01718	0.3595 *
TOTD “*PM Peak Hours*”	0.05746	0.01537	0.0002
TOTD “*Early Evening Hours*”	0.090575	0.01537	<0.0001
TOTD “*Late Evening Hours*”	0.146904	0.01437	<0.0001
Population density	−0.000105	0.00007	0.1459 *
Employment density	0.00089	0.00049	0.0734 *
Distance to city centre	0.007584	0.00411	0.068 *
Distance to the nearest bus stop	−0.065410	0.09992	0.5142 *
Road class “*Minor*”	−0.05799	0.03249	0.0772 *
Road length	−0.058438	0.025	0.0214
Connectivity of major road	0.027201	0.00923	0.004
Connectivity of minor road	0.041115	0.00964	<0.0001
Land use mix	−0.019881	0.02038	0.3316 *
DLUT “*Natural*”	0.043976	0.04296	0.3084 *
DLUT “*Other built-up*”	0.016395	0.02872	0.5693 *
DLUT “*Residential*”	0.060977	0.02482	0.0157
CTGS “*Yes*”	0.021779	0.02044	0.2891 *
Volume of motor vehicles	−0.000909	0.00035	0.0116
Traffic accident density	−14.31249	42.86109	0.7391 *
AIC	−493.1176		
BIC	−357.0646		
Restricted log-likelihood	269.5588		

Note: * means it is not statistically significant at a 0.05 level.
